# New Drug Updates in Hematologic Malignancies: CAR-T, Targeted Therapeutics, and Other Agents

**Published:** 2018-04-01

**Authors:** R. Donald Harvey

**Affiliations:** Emory University and Winship Cancer Institute, Atlanta, Georgia

## Abstract

FDA approvals for hematologic malignancies from 2016 to 2017 can be grouped in three themes: personalized small molecules, immunotherapies, and new formulations or modes of delivery for conventional agents. The unique profiles of these drugs, including that of CAR-T cell therapy, are summarized.

Newly approved drugs are changing outcomes for a number of hematologic malignancies. At JADPRO Live 2017, an update on 2017 drug approvals was presented by R. Donald Harvey, PharmD, BCOP, FCCP, FHOPA, Associate Professor of Hematology/Medical Oncology and Pharmacology and Director of the Phase I Clinical Trials Section at Emory University and the Winship Cancer Institute in Atlanta.

"It’s been a banner year for hematologic cancers in terms of drug approvals. We have personalized small molecules to hone in on subpopulations of patients, and we have ’sawed-off shotgun’ immunotherapies that teach the immune system to attack what should be foreign to the body: cancer," Dr. Harvey said. "The drugs that have been approved since 2016 are indicative of those two major paradigms."

Many are novel, first-in-class agents, while some are older drugs with new formulations or indications (Table 1). "Each agent has a unique profile and use criteria that should be reviewed prior to prescribing," Dr. Harvey emphasized.

**Table 1:**
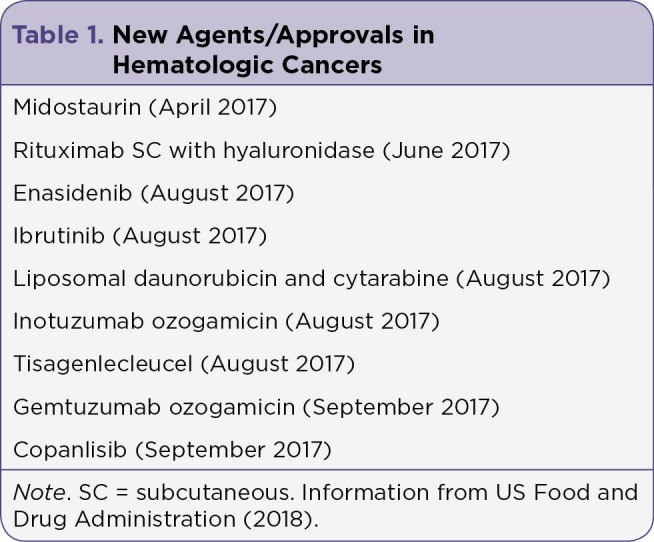
New Agents/Approvals in Hematologic Cancers

"We have three main buckets: immuno-oncology drugs, drugs that target particular populations, and new formulations or modes of delivery for conventional agents," he said.

## CAR T-CELL THERAPIES FOR LYMPHOMA

In 2017, tisagenlecleucel (Kymriah) became the first chimeric antigen receptor (CAR) T-cell therapeutic to be approved in any disease; it is indicated for patients up to age 25 with B-cell precursor acute lymphoblastic leukemia (ALL) that is refractory or in second or later relapse. This was followed by the approval of a second CAR T-cell product, axicabtagene ciloleucel (Yescarta), for adults with large B-cell lymphoma after progression on other therapies, including diffuse large B-cell lymphoma, primary mediastinal large B-cell lymphoma, high-grade B-cell lymphoma, and diffuse large B-cell lymphoma arising from follicular lymphoma.

CAR T-cell therapy is individualized immunotherapy based on the antigen of interest for a particular malignancy. The patient’s own T cells are engineered to display antigen receptors as "warheads" to hone in on and attack tumor cells when infused back into the patient. When T cells recognize their target, they are activated, leading to the release of cytokines, natural killer cells, cytotoxic T lymphocytes, and other effector components.

The challenge of these engineered cells is to avoid inhibitor and suppressive signals from the target cells, regulatory immune cells, and the tumor microenvironment. To create an environment where the CAR T cells will be welcomed, the patient undergoes lymphodepleting therapy with fludarabine and cyclophosphamide. A few days later, the T-cell product is transfused into the patient, where CD8+ and CD4+ cells will expand and persist until the tumor is eliminated. When successful, this process leads to long-term remission.

Tisagenlecleucel, an anti-CD19 CAR T-cell therapy, was approved based on a single-arm trial of 63 patients with relapsed or refractory pediatric precursor B-cell ALL, including 35 patients with prior hematopoietic stem cell transplant (Pulsipher et al., 2017). The overall remission rate was 82.5%, including 63% of patients with a complete response.

"This was in a young population, all with severe ALL, and 8 of 10 patients went into remission. That’s unheard of. It’s why there is excitement around this therapy," Dr. Harvey commented. "The efficacy comes with a price, but for the right patient population the payoff is worth the price."

This potential "price" can include cytokine release syndrome, a result of "cranking up" the immune system. Tocilizumab (Actemra), which is dosed according to weight, is approved for the treatment of this complication, he said. Other complications can include prolonged cytopenias, serious infections, and hypogammaglobulinemia.

## GEMTUZUMAB OZOGAMICIN FOR CD33+ AML

Gemtuzumab ozogamicin (Mylotarg) is an antibody-drug conjugate that was used to treat acute myeloid leukemia (AML) from 2000 to 2010. It was withdrawn from the market due to failure to confirm clinical benefit post-approval concerns. Subsequent studies with positive findings resulted in the resurrection of gemtuzumab ozogamicin and its approval in 2017. It is indicated as a first-line treatment for adults with CD33+ AML and for pediatric patients, 2 years of age and older, with relapsed or refractory CD33+ AML.

The phase III ALFA-0701 trial evaluated gemtuzumab ozogamicin in 280 newly diagnosed de novo AML patients, randomizing them to chemotherapy alone or chemotherapy plus gemtuzumab at a lower fractionated dose of 3 mg/m² on days 1, 4, and 7 (Castaigne et al., 2012). At 2 years, event-free survival was 17.1% in the control group vs. 40.8% in the gemtuzumab group (hazard ratio (HR), 0.58; *p* = .0003), and overall survival was 41.9% vs. 53.2%, respectively (HR, 0.69; *p* = .0368).

The dosing varies for de novo AML (combination regimens), newly diagnosed AML (single agent), and relapsed or refractory AML (single agent). Warnings and precautions include hepatotoxicity, infusion-related reactions, and hemorrhage, which can occur at recommended doses and can be severe.

"Gemtuzumab ozogamicin provides a nice addition to the armamentarium for AML," Dr. Harvey remarked.

## INOTUZUMAB OZOGAMICIN FOR PHILADELPHIA+ ALL

Similar to gemtuzumab ozogamicin is inotuzumab ozogamicin (Besponsa), an antibody/chemotherapy conjugate that internalizes into the tumor cells upon binding to CD22 on the cell surface. "It’s bringing a CD22 Trojan horse to the cell, releasing the payload there (the microtubule-targeting agent calicheamicin) so the cell is targeted for death," he explained.

"Inotuzumab looks promising in a number of lymphomas, but it came to market first for adults with relapsed or refractory B-cell precursor ALL," he said.

The pivotal multicenter phase III trial enrolled 326 patients with relapsed/refractory CD22+ ALL, randomizing them to a standard treatment or inotuzumab ozogamicin (Kantarjian et al., 2016). Complete remission rates were 80.7% with inotuzumab ozogamicin vs. 29.4% with standard therapy (*p* < .001). More patients in the inotuzumab arm had results below the threshold for minimal residual disease (MRD; 78.4% vs. 28.1%, *p* < .001). Median progression-free was significantly longer with inotuzumab (5.0 months vs. 1.8 months, *p* < .001), as was median overall survival (7.7 vs. 6.7 months, *p* = .04).

"Inotuzumab ozogamicin is therefore another option for adult patients with Philadelphia chromosome–positive ALL," he said.

Premedication is required before dosing. Warnings and precautions include hepatotoxicity/veno-occlusive disease or sinusoidal obstruction syndrome and increased risk of post-transplant nonrelapse mortality, myelosuppression, infusion reactions, and QT prolongation. Clinicians should monitor for liver function.

## IBRUTINIB IN CHRONIC GRAFT-VS.-HOST DISEASE

In 2017, ibrutinib (Imbruvica) became the first drug approved for chronic graft-vs.-host disease following allogeneic stem cell transplant. It is dosed at 420 mg daily, based on a trial of 42 patients who failed corticosteroids (Miklos et al., 2016). The overall response rate was 67%, median time to response was 12.3 weeks, and activity was seen in all involved organs.

## MIDOSTAURIN IN *FLT3*-MUTATED AML

Midostaurin (Rydapt) was approved for patients with newly diagnosed AML harboring *FLT3* mutations, in combination with standard cytarabine and daunorubicin induction and cytarabine consolidation. Midostaurin is a small molecule that inhibits wild-type and mutant FLT3 kinases as well as a number of other factors. It is given at 50 mg twice daily with food on days 8 to 21 of induction and consolidation chemotherapy; for maintenance, it is dosed continuously after consolidation "for as long as you can give it," he said.

Dr. Harvey recommended prophylactic antiemetics and watching for pneumonitis without an infectious cause. "A common theme for small-molecule tyrosine kinase inhibitors is the occurrence of noninfectious pneumonitis," he noted. "It’s generally straightforward, but you should hold the drug if it occurs." It should be discontinued in patients with signs of pulmonary toxicity. Grade 3/4 adverse reactions tend to be febrile neutropenia, device-related infection, and mucositis, which are already common with induction chemotherapy.

He also cautioned against giving midostaurin along with strong CYP3A inhibitors and inducers. "There are evolving data on how to think about azoles and midostaurin. Avoiding the azole is the best scenario, or you might take a bit of a risk but follow these patients closely."

A survival advantage, 23% reduced mortality risk, was seen in the phase III RATIFY trial of 717 newly diagnosed *FLT3*-mutated AML patients (*p* = .016; Schlenk et al., 2016). Thus, midostaurin plus conventional chemotherapy is the standard of care now in this population.

## ENASIDENIB FOR *IDH*-MUTATED AML

Mutations in isocitrate dehydrogenase (IDH) occur in 20% of AML cases and are also found in gliomas and cholangiocarcinomas. Isocitrate dehydrogenase regulates cellular metabolic fate; if it is inhibited, the cell cannot rid itself of endogenous products and is marked for apoptosis.

Enasidenib (Idhifa), an IDH2 inhibitor, is approved for relapsed or refractory AML patients with *IDH2* mutations as detected by the RealTime IDH2 Assay. Dosing is 100 mg orally once daily, continuously. There are no significant interactions with foods and other agents.

The enasidenib approval was based on Study AG221-C-001, an open-label, single-arm, multicenter trial that included 199 adults with relapsed or refractory AML with *IDH2* mutations. After a median follow-up time of 6.6 months, 23% of patients experienced a complete response or complete response with partial hematologic improvement (The ASCO Post, 2018).

Warnings and precautions include tumor lysis syndrome, differentiation syndrome (treated with hemodynamic monitoring, support, and steroids), leukocytosis, and bilirubin elevation more than three times the upper limit of normal (treated with dose adjustments).

"*IDH2* mutations tend to be seen in the elderly. Certainly, this is a challenging population. They may not be able to tolerate another induction regimen," Dr. Harvey said. "You can consider this drug, with careful monitoring for tumor lysis syndrome and differentiation syndrome."

## COPANLISIB FOR FOLLICULAR LYMPHOMA

Copanlisib (Aliqopa) was approved for adults with relapsed follicular lymphoma who have received at least two prior systemic therapies. It works by inhibiting the phosphatidylinositol 3-kinases (PI3Ks), PI3K-α and PI3K-δ, present in cancerous B cells. The PI3K pathway is involved in cell growth, survival, and metabolism, and its dysregulation plays an important role in follicular lymphoma. By blocking PI3K’s pathway, copanlisib inhibits tumor cell growth and survival.

It is given at 60 mg intravenously, weekly for 3 weeks on and 1 week off. Doses should be reduced to 45 mg in patients also taking strong CYP3A inhibitors, and the drug should be avoided in patients on strong CYP3A inducers.

Approval was based on data from the phase I single-arm CHRONOS-1 trial of 104 patients with follicular B-cell non-Hodgkin lymphoma who relapsed following at least two prior treatments (Dreyling et al., 2017). The overall response rate was to copanlisib was 59%, and median duration of response was 12.2 months.

"PI3K inhibitors have downstream effects of glucose trafficking," he noted. Patients can develop hyperglycemia, hypertension, neutropenia, severe rash, infections, and noninfectious pneumonitis.

## SUBCUTANEOUS RITUXIMAB WITH HYALURONIDASE

The presence of hyaluronidase allows larger volumes of drug to be injected into the subcutaneous space, making treatment more convenient. In 2017, rituximab was added to a number of drugs that can now be given subcutaneously (Rituxan Hycela).

Patients must receive one intravenous infusion before switching to subcutaneous rituximab. Depending on cancer type, rituximab is injected into the abdomen at a flat dose of 1,400 mg or 1,600 mg, with 23,400 or 26,800 units of hyaluronidase.

"Premedications remain the same, but you observe the patient for only 15 minutes. This is very different from our usual rituximab experience," Dr. Harvey said. "It’s an option for patients and institutions that want to give rituximab a little more quickly."

## LIPOSOMAL DAUNORUBICIN AND CYTARABINE

Liposomal daunorubicin and cytarabine (Vyxeos) is "not your grandmother’s 7 + 3," Dr. Harvey noted. This formulation delivers a higher concentration of drugs to the cells of interest and "improves the therapeutic index in hard-to-treat patients," he said.

The designation is for the treatment of adults with therapy-related AML or AML with myelodysplasia-related changes. The combination received a Breakthrough Therapy designation primarily based upon the positive results from the pivotal phase III clinical trial in older patients with previously untreated high-risk (secondary) AML in which the new regimen led to a median overall survival of 9.6 months, compared to 5.9 months for standard 7 + 3 therapy, a 31% reduction in risk (*p* = .005). At 12 months, overall survival was 41.5% vs. 27.5% (Lancet et al., 2016).

"The dosing is different, so talk to your pharmacist," he advised. For induction, daunorubicin is given at 44 mg/m² and cytarabine at 100 mg/m², over 90 minutes on days 1, 3, and 5 and on days 1 and 3 for subsequent cycles of induction if needed. Consolidation is with daunorubicin at 29 mg/m² and cytarabine at 65 mg/m² over 90 minutes on days 1 and 3. The warnings and precautions are the same as those for 7 + 3: cardiotoxicity, cytopenias, and extravasation.

"This is 7 + 3 in a different package," he said. "There are lots of options for using this compound in various AML settings."

## OTHER APPROVALS

Lenalidomide (Revlimid) can now be prescribed for maintenance therapy in multiple myeloma. A 200-mg dose of pembrolizumab (Keytruda) is approved for Hodgkin lymphoma. JAK2 testing is available now, to identify mutations associated with polycythemia vera. The oral anticoagulant betrixaban (Bevyxxa) can be prescribed for thromboembolism prophylaxis in medically ill patients. The label for blinatumomab (Blincyto) was expanded to include Philadelphia chromosome–positive acute lymphoblastic leukemia (not just negative).
